# Bis(2,2′-bipyrid­yl)-1κ^2^ *N*,*N*′;3κ^2^ *N*,*N*′-bis­(4-bromo-2-formyl­phenolato)-1κ^2^ *O*,*O*′;3κ^2^ *O*,*O*′-bis­[μ-2-(5-bromo-2-oxidobenzylidene­amino)­ethane­sul­fon­ato]-1:2κ^3^ *O*:*N*,*O* ^2^;2:3κ^3^ *N*,*O* ^2^:*O*-tricopper(II) monohydrate

**DOI:** 10.1107/S1600536809014263

**Published:** 2009-04-22

**Authors:** Ling Zhang

**Affiliations:** a Department of Chemistry, Lishui University, 323000 Lishui, Zhejiang, People’s Republic of China

## Abstract

The title complex, [Cu_3_(C_9_H_8_BrNO_4_S)_2_(C_7_H_4_BrO_2_)_2_(C_10_H_8_N_2_)_2_]·H_2_O, lies on an inversion center located on the central Cu atom, which is four-coordinated in a square-planar geometry, whereas the outer Cu atoms related by symmetry are five-coordinated in a square-pyramidal geometry. The trinuclear mol­ecules, with an intramolecular Cu⋯Cu separation of 6.313 (3) Å, are linked to each other, forming a chain through O—H⋯O and O—H⋯Br hydrogen bonds involving the half-occupied water mol­ecule. Futhermore, weak C—H⋯O inter­actions link the chains to form a supra­molecular network.

## Related literature

For general background on coordination polymers and open framework materials, see: Kim *et al.* (2003 ▶); Iglesias *et al.* (2003 ▶); Moulton & Zaworotko (2001 ▶). For background on 2,2′-bipyridyl and 5-bromo-2-hydroxy­benzaldehyde, see: Sun & Gao (2005 ▶); Murphy *et al.* (2004 ▶).
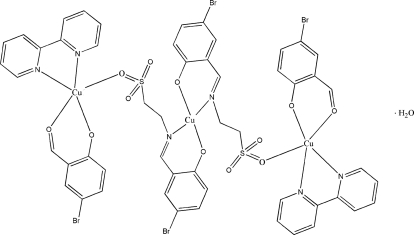


## Experimental

### 

#### Crystal data

[Cu_3_(C_9_H_8_BrNO_4_S)_2_(C_7_H_4_BrO_2_)_2_(C_10_H_8_N_2_)_2_]·H_2_O
*M* *_r_* = 1533.30
Triclinic, 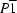

*a* = 10.031 (2) Å
*b* = 11.480 (2) Å
*c* = 12.913 (3) Å
α = 73.13 (3)°
β = 78.58 (3)°
γ = 75.24 (3)°
*V* = 1363.6 (6) Å^3^
*Z* = 1
Mo *K*α radiation
μ = 4.24 mm^−1^
*T* = 293 K
0.23 × 0.16 × 0.10 mm

#### Data collection

Bruker APEXII area-detector diffractometer
Absorption correction: multi-scan (*SADABS*; Bruker, 2007 ▶) *T*_min_ = 0.442, *T*_max_ = 0.677
12051 measured reflections
4888 independent reflections
1651 reflections with *I* > 2σ(*I*)
*R*_int_ = 0.077

#### Refinement

*R*[*F* ^2^ > 2σ(*F* ^2^)] = 0.047
*wR*(*F* ^2^) = 0.099
*S* = 0.76
4888 reflections
367 parameters
H-atom parameters constrained
Δρ_max_ = 0.43 e Å^−3^
Δρ_min_ = −0.39 e Å^−3^

### 

Data collection: *APEX2* (Bruker, 2007 ▶); cell refinement: *APEX2*; data reduction: *APEX2*; program(s) used to solve structure: *SHELXS97* (Sheldrick, 2008 ▶); program(s) used to refine structure: *SHELXL97* (Sheldrick, 2008 ▶); molecular graphics: *PLATON* (Spek, 2009 ▶); software used to prepare material for publication: *SHELXTL* (Sheldrick, 2008 ▶).

## Supplementary Material

Crystal structure: contains datablocks I, global. DOI: 10.1107/S1600536809014263/dn2446sup1.cif

Structure factors: contains datablocks I. DOI: 10.1107/S1600536809014263/dn2446Isup2.hkl

Additional supplementary materials:  crystallographic information; 3D view; checkCIF report

## Figures and Tables

**Table 1:** Hydrogen-bond geometry (Å, °)

*D*—H⋯*A*	*D*—H	H⋯*A*	*D*⋯*A*	*D*—H⋯*A*
O1*W*—H1*WB*⋯O2	0.84	2.40	3.197 (11)	159
O1*W*—H1*WA*⋯Br2^i^	0.83	2.55	3.145 (9)	130
C4—H4⋯O1^ii^	0.93	2.42	3.316 (9)	163
C23—H23⋯O2^iii^	0.93	2.54	3.324 (9)	142

Symmetry codes: (i) 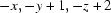; (ii) 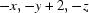; (iii) 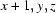.

**Fig. 1.**
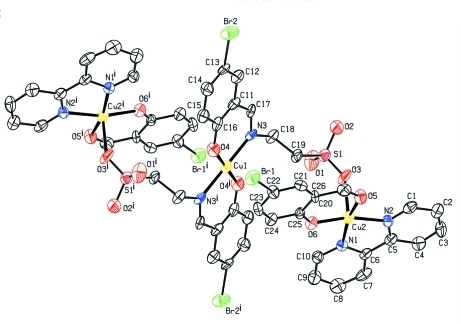
The structure of the trinuclear complex with the atom labeling scheme, Displacement ellipsoids are shown at the 30% probability level. H atom and water molecule have been omitted for clarity. [Symmetry code: (i) -x+1, -y+1, -z+1]

**Fig. 2.**
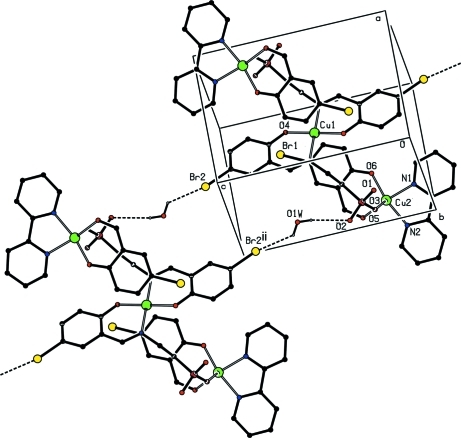
Partial packing view showing the H bond interactions linking the trinuclear unit through the water molecule. Hydrogen bonds are shown as dashed lines. H atoms not involved in hydrogen bonding have been omitted for clarity.[Symmetry codes: (ii) -x, -y+1, -z+2]

## supplementary crystallographic information

### Comment

Molecular self-assembly of supramolecular architectures has received much attention during recent decades (Kim *et al.*, 2003; Iglesias *et al.*, 2003; Moulton & Zaworotko, 2001). The structures and properties of such systems depend on the coordination and geometric preferences of both the central metals ions and bridging building blocks as well as the influence of weaker non-covalent interactions, such as hydrogen bonds and π-π stacking interactions. 2,2'-bipyridyl, 5-bromo-2-hydroxybenzaldehyde are excellent candidates for the construction of supramolecula complexes, since they not only have multiple coordination modes but also can form regular hydrogen bonds by functioning as both hydrogen-bond donor and acceptor (Sun & Gao, 2005; Murphy *et al.*, 2004). 2-(5-bromo-2-hydroxybenzylamino)ethanesulfonic has a versatile binding ability, whose structure of complexes have not been reported to date. Recently, we obtained the title novel trinuclear copper complex (I) by the reaction of copper nitryl, 2,2'-bipyridyl, 5-bromo-2-hydroxybenzaldehyde and 2-(5-bromo-2-hydroxybenzylamino)ethanesulfonic in an aqueous solution, and its crystal is reported here.

The trinuclear complex lyies on a crystallographic inversion center located on the central Cu1 atom which is four-coordinated in a square planar geometry, whereas the other Cu2 atoms related by symmetry are five-coordinated in a square pyramidal geometry (Fig. 1). The compound forms trinuclear structure *via* the flexible 2-(5-bromo-2-hydroxybenzylamino)ethanesulfonic ligand, with a Cu···Cu separation of 6.313 (3) Å. These trinuclear units are linked to each other to form a chain through O-H···O and O-H···Br hydrogen bonds involving the water molecule (table 1, Fig. 2). Futhermore, weak C-H···O interactions link the chain to form a supramolecular network.

### Experimental

A mixture of copper chloride(1 mmol), 5-bromo-2-hydroxybenzaldehyde (1 mmol), 2,2'-bipyridyl(1 mmol), 2-(5-bromo-2-hydroxybenzylamino)ethanesulfonic (1 mmol) and H_2_O (12 ml) was placed in a 23 ml Teflon reactor, which was heated to 433 K for three days and then cooled to room temperature at a rate of 10 K h^-1^. The crystals obtained were washed with water and dryed in air.

### Refinement

All H atoms attached to C atoms were fixed geometrically and treated as riding with C—H = 0.93 Å (aromatic) or 0.97 Å (methylene) with U_iso_(H) = 1.2U_eq_(C). H atoms of water molecule were located in difference Fourier maps and included in the subsequent refinement using  restraints (O-H= 0.82 (1)Å and H···H= 1.38 (2)Å) with U_iso_(H) = 1.5U_eq_(O). In the last stage of refinement, they were treated as riding on their parent O atom.

### Crystal data

**Table:**

[Cu_3_(C_9_H_8_BrNO_4_S)_2_(C_7_H_4_BrO_2_)_2_(C_10_H_8_N_2_)_2_]·H_2_O	*Z* = 1
*M**_r_* = 1533.30	*F*(000) = 759
Triclinic, *P*1	*D*_x_ = 1.867 Mg m^−^^3^
Hall symbol: -P 1	Mo *K*α radiation, λ = 0.71073 Å
*a* = 10.031 (2) Å	Cell parameters from 2895 reflections
*b* = 11.480 (2) Å	θ = 2.4–27.9°
*c* = 12.913 (3) Å	µ = 4.24 mm^−^^1^
α = 73.13 (3)°	*T* = 293 K
β = 78.58 (3)°	Block, colorless
γ = 75.24 (3)°	0.23 × 0.16 × 0.10 mm
*V* = 1363.6 (6) Å^3^	

### Data collection

**Table:**

Bruker APEXII area-detector diffractometer	4888 independent reflections
Radiation source: fine-focus sealed tube	1651 reflections with *I* > 2σ(*I*)
graphite	*R*_int_ = 0.077
φ and ω scans	θ_max_ = 25.2°, θ_min_ = 1.7°
Absorption correction: multi-scan (*SADABS*; Bruker, 2007)	*h* = −12→12
*T*_min_ = 0.442, *T*_max_ = 0.677	*k* = −13→13
12051 measured reflections	*l* = −15→15

### Refinement

**Table:**

Refinement on *F*^2^	Primary atom site location: structure-invariant direct methods
Least-squares matrix: full	Secondary atom site location: difference Fourier map
*R*[*F*^2^ > 2σ(*F*^2^)] = 0.047	Hydrogen site location: inferred from neighbouring sites
*wR*(*F*^2^) = 0.099	H-atom parameters constrained
*S* = 0.76	*w* = 1/[σ^2^(*F*_o_^2^) + (0.0382*P*)^2^] where *P* = (*F*_o_^2^ + 2*F*_c_^2^)/3
4888 reflections	(Δ/σ)_max_ = 0.001
367 parameters	Δρ_max_ = 0.43 e Å^−^^3^
0 restraints	Δρ_min_ = −0.39 e Å^−^^3^

### Special details

**Table:**

Geometry. All esds (except the esd in the dihedral angle between two l.s. planes) are estimated using the full covariance matrix. The cell esds are taken into account individually in the estimation of esds in distances, angles and torsion angles; correlations between esds in cell parameters are only used when they are defined by crystal symmetry. An approximate (isotropic) treatment of cell esds is used for estimating esds involving l.s. planes.
Refinement. Refinement of *F*^2^ against ALL reflections. The weighted *R*-factor *wR* and goodness of fit *S* are based on *F*^2^, conventional *R*-factors *R* are based on *F*, with *F* set to zero for negative *F*^2^. The threshold expression of *F*^2^ > σ(*F*^2^) is used only for calculating *R*-factors(gt) *etc*. and is not relevant to the choice of reflections for refinement. *R*-factors based on *F*^2^ are statistically about twice as large as those based on *F*, and *R*- factors based on ALL data will be even larger.

### Fractional atomic coordinates and isotropic or equivalent isotropic displacement parameters (Å^2^)

**Table:**

	*x*	*y*	*z*	*U*_iso_*/*U*_eq_	Occ. (<1)
Cu1	0.5000	0.5000	0.5000	0.0567 (4)	
Cu2	0.16643 (9)	1.01005 (8)	0.23932 (7)	0.0545 (3)	
Br1	0.56422 (9)	0.78255 (8)	0.70805 (6)	0.0780 (3)	
Br2	0.20594 (10)	0.35954 (9)	1.08489 (7)	0.0900 (4)	
S1	0.0325 (2)	0.7374 (2)	0.34555 (18)	0.0589 (6)	
N1	0.2317 (7)	0.9972 (6)	0.0858 (5)	0.0490 (16)	
N2	0.0114 (6)	1.1303 (5)	0.1664 (6)	0.0499 (17)	
N3	0.3009 (6)	0.5065 (5)	0.5526 (5)	0.0539 (18)	
O1	0.0575 (6)	0.6616 (5)	0.2703 (4)	0.100 (2)	
O2	−0.0917 (5)	0.7223 (5)	0.4217 (4)	0.0901 (18)	
O3	0.0319 (5)	0.8680 (4)	0.2953 (4)	0.0853 (18)	
O4	0.5413 (5)	0.4760 (5)	0.6422 (4)	0.0695 (16)	
O5	0.1069 (5)	1.0634 (4)	0.3756 (4)	0.0677 (16)	
O6	0.3395 (4)	0.9154 (4)	0.2812 (3)	0.0529 (13)	
C1	−0.0981 (10)	1.1957 (8)	0.2182 (6)	0.066 (2)	
H1	−0.1040	1.1861	0.2929	0.079*	
C2	−0.2050 (8)	1.2792 (7)	0.1604 (8)	0.070 (2)	
H2	−0.2822	1.3230	0.1965	0.084*	
C3	−0.1923 (10)	1.2938 (8)	0.0521 (8)	0.078 (3)	
H3	−0.2616	1.3488	0.0131	0.094*	
C4	−0.0822 (9)	1.2309 (8)	−0.0011 (7)	0.066 (3)	
H4	−0.0750	1.2429	−0.0762	0.080*	
C5	0.0231 (9)	1.1459 (7)	0.0577 (8)	0.056 (2)	
C6	0.1483 (9)	1.0735 (7)	0.0102 (7)	0.054 (2)	
C7	0.1860 (10)	1.0791 (7)	−0.0994 (7)	0.070 (3)	
H7	0.1294	1.1312	−0.1507	0.083*	
C8	0.3088 (11)	1.0058 (9)	−0.1304 (7)	0.074 (3)	
H8	0.3363	1.0089	−0.2041	0.089*	
C9	0.3919 (9)	0.9284 (7)	−0.0566 (8)	0.074 (3)	
H9	0.4750	0.8777	−0.0781	0.089*	
C10	0.3483 (9)	0.9282 (7)	0.0509 (7)	0.059 (2)	
H10	0.4047	0.8760	0.1023	0.071*	
C11	0.3250 (10)	0.4410 (6)	0.7469 (6)	0.059 (2)	
C12	0.2482 (8)	0.4121 (6)	0.8509 (6)	0.059 (2)	
H12	0.1554	0.4079	0.8578	0.071*	
C13	0.3108 (9)	0.3899 (6)	0.9436 (5)	0.058 (2)	
C14	0.4491 (9)	0.3968 (7)	0.9313 (7)	0.069 (2)	
H14	0.4903	0.3812	0.9939	0.083*	
C15	0.5277 (8)	0.4253 (6)	0.8325 (7)	0.061 (2)	
H15	0.6201	0.4296	0.8276	0.073*	
C16	0.4641 (9)	0.4487 (7)	0.7348 (7)	0.056 (2)	
C17	0.2491 (7)	0.4750 (5)	0.6544 (6)	0.048 (2)	
H17	0.1547	0.4741	0.6694	0.057*	
C18	0.1956 (7)	0.5436 (6)	0.4753 (5)	0.056 (2)	
H18A	0.1090	0.5212	0.5143	0.067*	
H18B	0.2284	0.4991	0.4186	0.067*	
C19	0.1708 (7)	0.6813 (6)	0.4242 (5)	0.058 (2)	
H19A	0.2551	0.7010	0.3781	0.070*	
H19B	0.1517	0.7245	0.4816	0.070*	
C20	0.3051 (8)	0.9405 (7)	0.4636 (6)	0.0427 (19)	
C21	0.3588 (8)	0.9097 (6)	0.5629 (6)	0.052 (2)	
H21	0.3079	0.9415	0.6208	0.062*	
C22	0.4862 (9)	0.8328 (7)	0.5734 (6)	0.053 (2)	
C23	0.5646 (8)	0.7859 (6)	0.4863 (7)	0.058 (2)	
H23	0.6518	0.7343	0.4951	0.069*	
C24	0.5151 (8)	0.8146 (6)	0.3881 (5)	0.051 (2)	
H24	0.5683	0.7829	0.3309	0.061*	
C25	0.3833 (8)	0.8923 (6)	0.3751 (7)	0.0455 (19)	
C26	0.1759 (9)	1.0241 (7)	0.4569 (6)	0.067 (2)	
H26	0.1361	1.0541	0.5181	0.080*	
O1W	−0.0713 (9)	0.6212 (10)	0.6775 (8)	0.101 (4)	0.50
H1WA	−0.1452	0.6481	0.7125	0.152*	0.50
H1WB	−0.0587	0.6569	0.6109	0.152*	0.50

### Atomic displacement parameters (Å^2^)

**Table:**

	*U*^11^	*U*^22^	*U*^33^	*U*^12^	*U*^13^	*U*^23^
Cu1	0.0656 (10)	0.0561 (9)	0.0502 (9)	−0.0107 (8)	−0.0282 (8)	−0.0044 (7)
Cu2	0.0598 (7)	0.0578 (7)	0.0444 (6)	−0.0086 (5)	−0.0168 (5)	−0.0072 (5)
Br1	0.0910 (7)	0.0913 (7)	0.0530 (6)	−0.0181 (6)	−0.0290 (5)	−0.0083 (5)
Br2	0.1119 (8)	0.1085 (8)	0.0554 (6)	−0.0490 (7)	−0.0213 (6)	−0.0006 (5)
S1	0.0574 (16)	0.0591 (17)	0.0571 (15)	−0.0124 (13)	−0.0245 (13)	0.0013 (13)
N1	0.057 (5)	0.045 (4)	0.049 (4)	−0.012 (4)	−0.015 (4)	−0.012 (4)
N2	0.045 (5)	0.050 (5)	0.057 (5)	−0.010 (4)	−0.012 (4)	−0.014 (4)
N3	0.074 (5)	0.052 (4)	0.037 (4)	−0.019 (4)	−0.028 (4)	0.005 (3)
O1	0.132 (5)	0.107 (5)	0.067 (4)	0.007 (4)	−0.059 (4)	−0.029 (4)
O2	0.059 (4)	0.080 (4)	0.116 (5)	−0.014 (3)	−0.006 (4)	−0.005 (4)
O3	0.083 (4)	0.060 (4)	0.102 (4)	−0.023 (3)	−0.058 (3)	0.033 (3)
O4	0.061 (4)	0.094 (4)	0.055 (4)	−0.011 (3)	−0.022 (3)	−0.018 (3)
O5	0.068 (4)	0.086 (4)	0.045 (3)	0.003 (3)	−0.016 (3)	−0.021 (3)
O6	0.061 (3)	0.059 (3)	0.044 (3)	−0.011 (3)	−0.019 (3)	−0.013 (3)
C1	0.077 (7)	0.069 (7)	0.054 (6)	−0.028 (6)	−0.016 (6)	−0.004 (5)
C2	0.063 (6)	0.057 (6)	0.090 (7)	−0.014 (5)	−0.024 (6)	−0.010 (6)
C3	0.070 (7)	0.075 (7)	0.085 (8)	0.008 (6)	−0.041 (6)	−0.013 (6)
C4	0.060 (6)	0.089 (7)	0.058 (6)	−0.016 (6)	−0.026 (6)	−0.016 (6)
C5	0.060 (7)	0.048 (6)	0.064 (7)	−0.020 (5)	−0.025 (6)	−0.004 (5)
C6	0.064 (7)	0.043 (6)	0.057 (6)	−0.015 (5)	−0.017 (6)	−0.006 (5)
C7	0.103 (8)	0.062 (7)	0.040 (6)	−0.016 (6)	−0.017 (6)	−0.003 (5)
C8	0.109 (9)	0.076 (7)	0.043 (6)	−0.032 (6)	0.007 (6)	−0.025 (6)
C9	0.083 (7)	0.068 (7)	0.067 (7)	−0.007 (5)	−0.013 (6)	−0.016 (6)
C10	0.052 (6)	0.066 (6)	0.054 (6)	−0.004 (5)	−0.013 (5)	−0.009 (5)
C11	0.085 (7)	0.058 (6)	0.035 (5)	−0.014 (5)	−0.022 (5)	−0.005 (4)
C12	0.075 (6)	0.047 (5)	0.061 (6)	−0.014 (5)	−0.022 (5)	−0.013 (5)
C13	0.093 (7)	0.048 (5)	0.032 (5)	−0.021 (5)	−0.015 (5)	0.001 (4)
C14	0.061 (6)	0.086 (7)	0.064 (6)	−0.016 (5)	−0.024 (5)	−0.014 (5)
C15	0.062 (6)	0.063 (6)	0.063 (6)	−0.013 (5)	−0.025 (5)	−0.012 (5)
C16	0.063 (7)	0.047 (6)	0.060 (7)	−0.001 (5)	−0.027 (6)	−0.011 (5)
C17	0.051 (5)	0.027 (5)	0.068 (6)	−0.002 (4)	−0.033 (5)	−0.004 (4)
C18	0.065 (5)	0.048 (5)	0.062 (5)	−0.018 (4)	−0.040 (4)	0.001 (4)
C19	0.061 (6)	0.051 (5)	0.056 (5)	−0.007 (4)	−0.031 (4)	0.008 (4)
C20	0.037 (5)	0.041 (5)	0.052 (6)	0.002 (4)	−0.011 (5)	−0.021 (4)
C21	0.052 (6)	0.051 (6)	0.054 (6)	−0.016 (5)	0.001 (5)	−0.019 (4)
C22	0.057 (6)	0.059 (6)	0.047 (5)	−0.013 (5)	−0.019 (5)	−0.011 (4)
C23	0.049 (6)	0.047 (5)	0.074 (6)	0.004 (4)	−0.017 (5)	−0.017 (5)
C24	0.062 (6)	0.050 (5)	0.044 (5)	−0.010 (5)	−0.018 (5)	−0.014 (4)
C25	0.046 (6)	0.035 (5)	0.055 (6)	−0.004 (4)	−0.014 (5)	−0.009 (4)
C26	0.085 (7)	0.069 (6)	0.046 (6)	−0.014 (6)	0.003 (5)	−0.025 (5)
O1W	0.071 (8)	0.148 (11)	0.085 (8)	0.006 (7)	0.019 (7)	−0.076 (8)

### Geometric parameters (Å, °)

**Table:**

Cu1—O4	1.889 (5)	C7—H7	0.9300
Cu1—O4^i^	1.889 (5)	C8—C9	1.356 (9)
Cu1—N3	1.967 (6)	C8—H8	0.9300
Cu1—N3^i^	1.967 (6)	C9—C10	1.370 (9)
Cu2—O6	1.886 (4)	C9—H9	0.9300
Cu2—O5	1.963 (5)	C10—H10	0.9300
Cu2—N2	1.986 (6)	C11—C16	1.395 (10)
Cu2—N1	1.996 (6)	C11—C12	1.403 (9)
Cu2—O3	2.249 (5)	C11—C17	1.449 (8)
Br1—C22	1.922 (7)	C12—C13	1.389 (8)
Br2—C13	1.901 (7)	C12—H12	0.9300
S1—O1	1.431 (5)	C13—C14	1.385 (9)
S1—O2	1.441 (5)	C14—C15	1.359 (9)
S1—O3	1.449 (4)	C14—H14	0.9300
S1—C19	1.758 (6)	C15—C16	1.450 (9)
N1—C10	1.314 (8)	C15—H15	0.9300
N1—C6	1.369 (8)	C17—H17	0.9300
N2—C1	1.341 (8)	C18—C19	1.502 (7)
N2—C5	1.348 (8)	C18—H18A	0.9700
N3—C17	1.296 (7)	C18—H18B	0.9700
N3—C18	1.495 (7)	C19—H19A	0.9700
O4—C16	1.292 (8)	C19—H19B	0.9700
O5—C26	1.280 (7)	C20—C26	1.404 (9)
O6—C25	1.303 (7)	C20—C21	1.404 (8)
C1—C2	1.415 (9)	C20—C25	1.421 (9)
C1—H1	0.9300	C21—C22	1.362 (8)
C2—C3	1.343 (9)	C21—H21	0.9300
C2—H2	0.9300	C22—C23	1.400 (9)
C3—C4	1.339 (9)	C23—C24	1.373 (8)
C3—H3	0.9300	C23—H23	0.9300
C4—C5	1.416 (9)	C24—C25	1.405 (8)
C4—H4	0.9300	C24—H24	0.9300
C5—C6	1.449 (10)	C26—H26	0.9300
C6—C7	1.379 (9)	O1W—H1WA	0.8251
C7—C8	1.363 (9)	O1W—H1WB	0.8381
			
O4—Cu1—O4^i^	180.000 (1)	C8—C9—H9	121.4
O4—Cu1—N3	92.1 (2)	C10—C9—H9	121.4
O4^i^—Cu1—N3	87.9 (2)	N1—C10—C9	124.0 (8)
O4—Cu1—N3^i^	87.9 (2)	N1—C10—H10	118.0
O4^i^—Cu1—N3^i^	92.1 (2)	C9—C10—H10	118.0
N3—Cu1—N3^i^	180.0 (3)	C16—C11—C12	120.9 (7)
O6—Cu2—O5	93.61 (19)	C16—C11—C17	122.1 (7)
O6—Cu2—N2	166.7 (2)	C12—C11—C17	116.7 (8)
O5—Cu2—N2	93.4 (3)	C13—C12—C11	120.0 (7)
O6—Cu2—N1	90.5 (2)	C13—C12—H12	120.0
O5—Cu2—N1	166.5 (2)	C11—C12—H12	120.0
N2—Cu2—N1	80.3 (3)	C14—C13—C12	119.0 (7)
O6—Cu2—O3	102.37 (17)	C14—C13—Br2	120.3 (6)
O5—Cu2—O3	91.9 (2)	C12—C13—Br2	120.7 (7)
N2—Cu2—O3	88.68 (19)	C15—C14—C13	123.3 (7)
N1—Cu2—O3	99.8 (2)	C15—C14—H14	118.4
O1—S1—O2	111.7 (4)	C13—C14—H14	118.4
O1—S1—O3	114.7 (3)	C14—C15—C16	118.5 (7)
O2—S1—O3	110.9 (3)	C14—C15—H15	120.7
O1—S1—C19	106.6 (3)	C16—C15—H15	120.7
O2—S1—C19	105.8 (3)	O4—C16—C11	124.6 (7)
O3—S1—C19	106.4 (3)	O4—C16—C15	117.0 (8)
C10—N1—C6	118.0 (7)	C11—C16—C15	118.3 (8)
C10—N1—Cu2	126.4 (6)	N3—C17—C11	125.8 (7)
C6—N1—Cu2	115.5 (6)	N3—C17—H17	117.1
C1—N2—C5	120.3 (7)	C11—C17—H17	117.1
C1—N2—Cu2	124.1 (6)	N3—C18—C19	110.6 (5)
C5—N2—Cu2	115.6 (6)	N3—C18—H18A	109.5
C17—N3—C18	113.8 (6)	C19—C18—H18A	109.5
C17—N3—Cu1	124.5 (5)	N3—C18—H18B	109.5
C18—N3—Cu1	121.6 (4)	C19—C18—H18B	109.5
S1—O3—Cu2	143.6 (3)	H18A—C18—H18B	108.1
C16—O4—Cu1	129.1 (5)	C18—C19—S1	114.0 (4)
C26—O5—Cu2	124.1 (5)	C18—C19—H19A	108.7
C25—O6—Cu2	127.8 (5)	S1—C19—H19A	108.7
N2—C1—C2	120.6 (8)	C18—C19—H19B	108.7
N2—C1—H1	119.7	S1—C19—H19B	108.7
C2—C1—H1	119.7	H19A—C19—H19B	107.6
C3—C2—C1	118.5 (9)	C26—C20—C21	116.3 (8)
C3—C2—H2	120.7	C26—C20—C25	123.4 (7)
C1—C2—H2	120.7	C21—C20—C25	120.3 (7)
C4—C3—C2	121.5 (9)	C22—C21—C20	119.2 (7)
C4—C3—H3	119.3	C22—C21—H21	120.4
C2—C3—H3	119.3	C20—C21—H21	120.4
C3—C4—C5	119.7 (8)	C21—C22—C23	120.9 (7)
C3—C4—H4	120.1	C21—C22—Br1	121.7 (6)
C5—C4—H4	120.1	C23—C22—Br1	117.4 (7)
N2—C5—C4	119.3 (8)	C24—C23—C22	121.2 (7)
N2—C5—C6	115.3 (8)	C24—C23—H23	119.4
C4—C5—C6	125.3 (9)	C22—C23—H23	119.4
N1—C6—C7	121.1 (8)	C23—C24—C25	119.3 (7)
N1—C6—C5	113.3 (8)	C23—C24—H24	120.3
C7—C6—C5	125.6 (9)	C25—C24—H24	120.3
C8—C7—C6	117.9 (8)	O6—C25—C24	117.2 (7)
C8—C7—H7	121.0	O6—C25—C20	123.7 (7)
C6—C7—H7	121.0	C24—C25—C20	119.1 (7)
C9—C8—C7	121.8 (9)	O5—C26—C20	127.0 (7)
C9—C8—H8	119.1	O5—C26—H26	116.5
C7—C8—H8	119.1	C20—C26—H26	116.5
C8—C9—C10	117.1 (8)	H1WA—O1W—H1WB	116.5

Symmetry codes: (i) −*x*+1, −*y*+1, −*z*+1.

### Hydrogen-bond geometry (Å, °)

**Table:**

*D*—H···*A*	*D*—H	H···*A*	*D*···*A*	*D*—H···*A*
O1W—H1WB···O2	0.84	2.40	3.197 (11)	159
O1W—H1WA···Br2^ii^	0.83	2.55	3.145 (9)	130
C4—H4···O1^iii^	0.93	2.42	3.316 (9)	163
C23—H23···O2^iv^	0.93	2.54	3.324 (9)	142

Symmetry codes: (ii) −*x*, −*y*+1, −*z*+2; (iii) −*x*, −*y*+2, −*z*; (iv) *x*+1, *y*, *z*.
